# Supplementary material for: Childhood intelligence and risk of depression in later-life: A longitudinal data-linkage study
Source: SSM Popul Health. 2023 Nov 17;25:101560. doi: 10.1016/j.ssmph.2023.101560 (PMC10709490; doi:10.1016/j.ssmph.2023.101560)
Supplement: Multimedia component 1 [file mmc1.docx]

**Childhood intelligence and risk of depression in later-life: A longitudinal data-linkage study**

**Supplementary material**

**Supplementary Table S1:** Table of ICD codes for depression

| **Code** | **Version** | **Code** | **Version** |
| --- | --- | --- | --- |
| -29620 | icd9 | F320 | icd10 |
| -29621 | icd9 | F321 | icd10 |
| -29622 | icd9 | F322 | icd10 |
| -29623 | icd9 | F323 | icd10 |
| -29624 | icd9 | F324 | icd10 |
| -29625 | icd9 | F325 | icd10 |
| -29626 | icd9 | F328 | icd10 |
| -29630 | icd9 | F329 | icd10 |
| -29631 | icd9 | F330 | icd10 |
| -29632 | icd9 | F331 | icd10 |
| -29633 | icd9 | F332 | icd10 |
| -29634 | icd9 | F333 | icd10 |
| -29635 | icd9 | F334 | icd10 |
| -29636 | icd9 | F338 | icd10 |
| -29690 | icd9 | F339 | icd10 |
| -29699 | icd9 | F340 | icd10 |
| -3004 | icd9 | F341 | icd10 |
| -30112 | icd9 | F348 | icd10 |
| -3090 | icd9 | F349 | icd10 |
| -3091 | icd9 | F380 | icd10 |
| -3110 | icd9 | F381 | icd10 |
| -3111 | icd9 | F388 | icd10 |
| -3112 | icd9 | F39X | icd10 |
| -3113 | icd9 | F4320 | icd10 |
| -3114 | icd9 | F4321 | icd10 |
| -3115 | icd9 |  |  |
| -3116 | icd9 |  |  |
| -3117 | icd9 |  |  |
| -3118 | icd9 |  |  |
| -3119 | icd9 |  |  |
| -2962 | icd9 |  |  |
| -2963 | icd9 |  |  |
| -311 | icd9 |  |  |
| -309 | icd9 |  |  |

**Supplementary Table S2:** Table of antidepressants

| **Approved name** |
| --- |
| AGOMELATINE |
| AMITRIPTYLINE |
| AMITRIPTYLINE HYDROCHLORIDE WITH PERPHENAZINE |
| CITALOPRAM |
| CLOMIPRAMINE HYDROCHLORIDE |
| DOSULEPIN HYDROCHLORIDE |
| DOXEPIN |
| DULOXETINE |
| ESCITALOPRAM |
| FLUOXETINE |
| FLUPENTIXOL |
| FLUVOXAMINE MALEATE |
| IMIPRAMINE HYDROCHLORIDE |
| LOFEPRAMINE |
| MIANSERIN HYDROCHLORIDE |
| MIRTAZAPINE |
| MOCLOBEMIDE |
| NORTRIPTYLINE |
| PAROXETINE |
| PHENELZINE |
| REBOXETINE |
| SERTRALINE |
| TRAZODONE HYDROCHLORIDE |
| TRIMIPRAMINE |
| TRYPTOPHAN |
| VENLAFAXINE |
| VORTIOXETINE HYDROBROMIDE |

**Supplementary Figure S3:** How many people had depression ICD codes reported in hospital admissions as the ‘main’ reason for admission and/or as an ‘other’ diagnosis?

|  |
| --- |
| 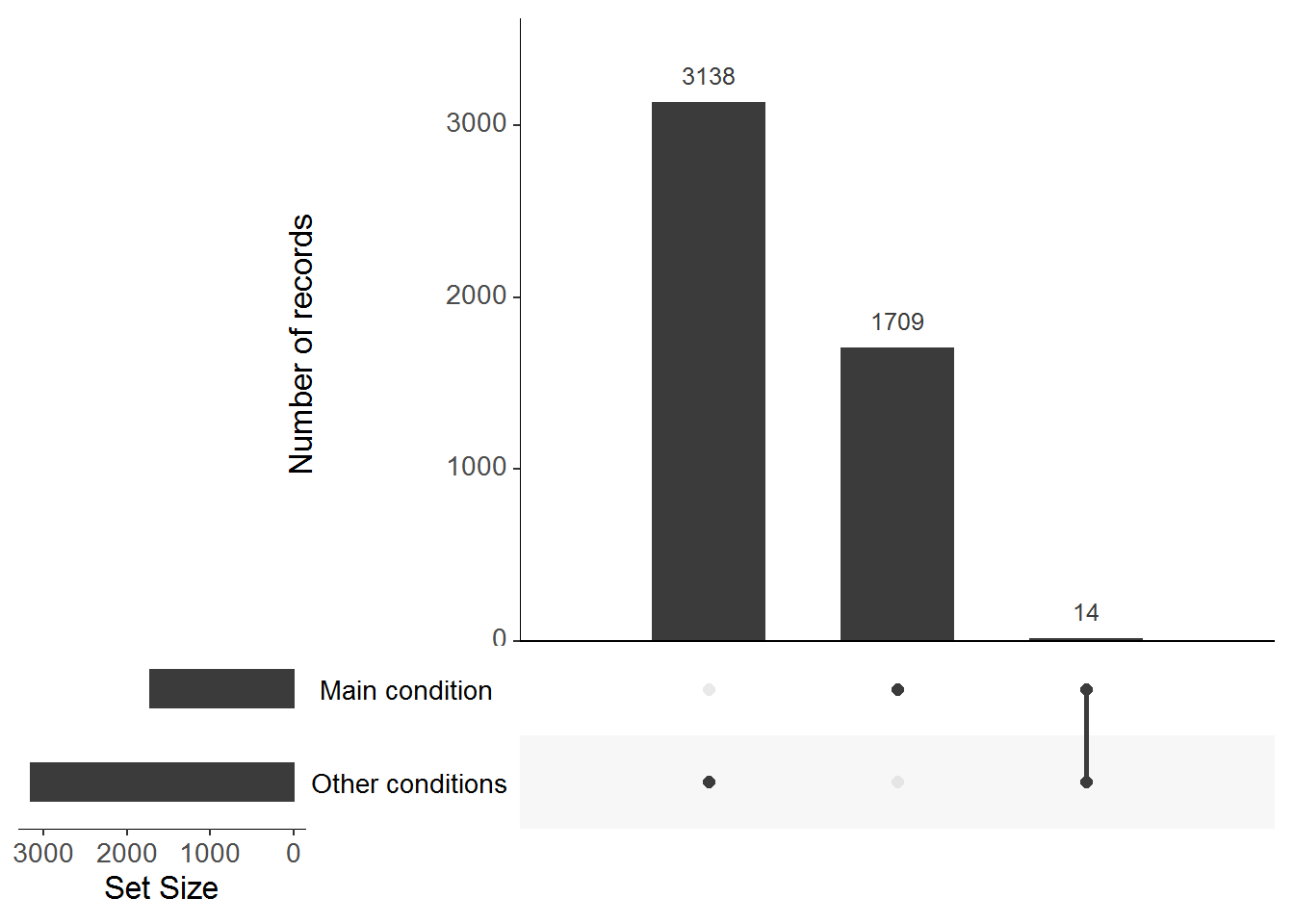 |
| The plot shows the number of hospital admission records that had depression reported as the ‘main’ reason for a hospital admission, and/or depression reported as an ‘other’ condition when a participant was admitted to hospital. The dots show whether the diagnosis of depression was reported as a main and/or other condition. All hospital admission records that report diagnosis of depression are shown in the plot (i.e., one participant could have multiple hospital admissions). |
|  |

| **Supplementary Table S4:** Risk factors associated with depression after unadjusted and adjusted analyses | | | | | | | | |
| --- | --- | --- | --- | --- | --- | --- | --- | --- |
|  | **Unadjusted risk factors**  **(Depression N/Total N) ±** | | **Model 1:**  **Adjusted for childhood**  **risk factors** | | **Model 2:**  **Adjusted for adulthood**  **risk factors** | | **Model 3:**  **Adjusted for all**  **risk factors** | |
|  | **CPH**  HR  (95% CI) | **Mixed effects model** | **CPH**  HR  (95% CI) | **Mixed effects model** | **CPH**  HR  (95% CI) | **Mixed effects**  **model** | **CPH**  HR  (95% CI) | **Mixed effects model** |
| **Depression N/Total N (%)±** | N/A | N/A | 13022/49151  (26%) | 16595/96102  (17%) | 2776/11702 (24%) | 5637/57341  (10%) | 2757/11617  (24%) | 5537/56771  (10%) |
| **Male** | 0.65  (0.63 to 0.68)  P<0.001  (n=14063/53037) | 0.68  (0.66 to 0.71)  P<0.001  (n=18043/104762) | 0.64  (0.62 to 0.67)  P<0.001 | 0.68  (0.65 to 0.71)  P<0.001 | 0.71  (0.66 to 0.77)  P<0.001 | 0.84  (0.77 to 0.92)  P<0.001 | 0.71  (0.66 to 0.77)  P<0.001 | 0.84  (0.77 to 0.92)  P<0.001 |
| **Moray House Test** | 0.89  (0.87 to 0.90)  P<0.001  (n=13118/49506) | 0.95  (0.93 to 0.97)  P<0.001  (n=16781/96960) | 0.88  (0.86 to 0.89)  P<0.001 | 0.94  (0.92 to 0.96)  P<0.001 | 0.91  (0.88 to 0.95)  P<0.001 | 0.95  (0.91 to 1.00)  P=0.035 | 0.92  (0.88 to 0.95)  P<0.001 | 0.95  (0.91 to 1.00)  P=0.032 |
| **Position in family** | 1.03  (1.02 to 1.03)  P<0.001  (n=13934/52542) | 1.01  (1.00 to 1.02)  P=0.142  (n=17813/103605) | N/A | N/A | N/A | N/A | N/A | N/A |
| **Size of family** | 1.07  (1.05 to 1.09)  P<0.001  (n=13931/52538) | 1.03  (1.01 to 1.05)  P=0.012  (n=17810/103591) | 1.02  (1.00 to 1.04)  P=0.013 | 1.00  (0.98 to 1.03)  P=0.709 | N/A | N/A | 1.02  (0.98 to 1.06)  P=0.338 | 1.00  (0.96 to 1.05)  P=1.000 |
| **Carstairs** | 1.03  (1.01 to 1.04)  P<0.001  (n=3047/12760) | 1.01  (1.00 to 1.03)  P=0.048  (6165/63163) | N/A | N/A | 1.02  (1.01 to 1.03)  P=0.006 | 1.01  (1.00 to 1.03)  P=0.200 | 1.02  (1.00 to 1.03)  P=0.008 | 1.01  (0.99 to 1.03)  P=0.252 |
| **Remote location*** | 0.92  (0.83 to 1.01)  P=0.088  (n=3022/12650) | 1.05  (0.94 to 1.18)  P=0.350  (n=6117/62376) | N/A | N/A | 0.98  (0.88 to 1.09)  P=0.722 | 1.09  (0.97 to 1.22)  P=0.165 | 0.98  (0.89 to 1.09)  P=0.769 | 1.09  (0.97 to 1.22)  P=0.160 |
| **SIMD – Linear** | 1.32  (1.18 to 1.49)  P<0.001  (n=3029/12692) | 1.12  (0.99 to 1.28)  P=0.077  (n=6127/62649) | N/A | N/A | N/A | N/A | N/A | N/A |
| CI: confidence interval; CPH: cox proportional hazards; HR: hazards ratio; SIMD: Scottish Index of Multiple Deprivation  *Accessible Rural Areas, Remote Rural Areas, Very Remote Rural Areas  ± CPH analyses is based on number of people in analysis, whereas, mixed effects models are based on number of observations  Cox proportional hazards: one record for each participant. The earliest diagnosis of depression. When a person had a hospital admission(s) for depression and were prescribed antidepressants, we included the earliest diagnosis of depression that had adult environmental factors reported.  The number of records included in each statistical model varies because records with missing environmental factors are excluded from the analyses. | | | | | | | | |

**Supplementary Table S5:** Sex stratified analyses

| **Supplement 5: Risk factors associated with depression after sex-stratified analyses** | | | | | | | | |
| --- | --- | --- | --- | --- | --- | --- | --- | --- |
|  | **Sex-stratified**  **risk factors**  **(Depression N/Total N) ±** | | **Model 1:**  **Adjusted for childhood**  **risk factors** | | **Model 2:**  **Adjusted for adulthood**  **risk factors** | | **Model 3:**  **Adjusted for all**  **risk factors** | |
|  | **CPH**  HR  (95% CI) | **Mixed effects model** | **CPH**  HR  (95% CI) | **Mixed effects model** | **CPH**  HR  (95% CI) | **Mixed effects**  **model** | **CPH**  HR  (95% CI) | **Mixed effects model** |
| **Depression N/Total N (%)±** | N/A | N/A | 13022/49151  (26%) | 16595/96102  (17%) | 2776/11702  (24%) | 5637/57341 (10%) | 2757/11617  (24%) | 5537/56771  (10%) |
| **Moray House Test** | 0.87  (0.86 to 0.89)  P<0.001  (n=13118/49506) | 0.94  (0.92 to 0.96)  P<0.001  (n=16781/96960) | 0.88  (0.86 to 0.89)  P<0.001 | 0.94  (0.92 to 0.96)  P<0.001 | 0.91  (0.88 to 0.95)  P<0.001 | 0.95  (0.91 to 1.00)  P=0.035 | 0.92  (0.88 to 0.96)  P<0.001 | 0.95  (0.91 to 1.00)  P=0.032 |
| **Position in family** | 1.02  (1.01 to 1.03)  P<0.001  (n=13934/52542) | 1.01  (1.00 to 1.02)  P=0.271  (n=17813/103605) | N/A | N/A | N/A | N/A | N/A | N/A |
| **Size of family** | 1.06  (1.05 to 1.08)  P<0.001  (n=13931/52538) | 1.02  (1.00 to 1.04)  P=0.026  (n=17810/103591) | 1.02  (1.00 to 1.04)  P=0.013 | 1.00  (0.98 to 1.03)  P=0.709 | N/A | N/A | 1.02  (0.98 to 1.06)  P=0.334 | 1.00  (0.96 to 1.05)  P=1.000 |
| **Carstairs** | 1.03  (1.01 to 1.04)  P<0.001  (n=3047/12760) | 1.01  (1.00 to 1.03)  P=0.044  (n=6165/63163) | N/A | N/A | 1.02  (1.01 to 1.03)  P=0.006 | 1.01  (1.00 to 1.03)  P=0.193 | 1.02  (1.00 to 1.03)  P=0.008 | 1.01  (0.99 to 1.03)  P=0.246 |
| **Remote location*** | 0.92  (0.84 to 1.02)  P=0.118  (n=3022/12650) | 1.06  (0.95 to 1.18)  P=0.289  (n=6117/62376) | N/A | N/A | 0.98  (0.88 to 1.09)  P=0.712 | 1.09  (0.97 to 1.22)  P=0.154 | 0.98  (0.88 to 1.09)  P=0.760 | 1.09  (0.97 to 1.22)  P=0.145 |
| **SIMD – Linear** | 1.35  (1.20 to 1.52)  P<0.001  (n=3029/12692) | 1.13  (1.00 to 1.28)  P=0.058  (n=6127/62649) | N/A | N/A | N/A | N/A | N/A | N/A |
| CI: confidence interval; CPH: cox proportional hazards; HR: hazards ratio  ± CPH analyses is based on number of people in analysis, whereas, mixed effects models are based on number of observations  *Accessible Rural Areas, Remote Rural Areas, Very Remote Rural Areas | | | | | | | | |

| **Supplementary Table S6:** Unadjusted mixed effect models performed on separate populations of females and males | | |
| --- | --- | --- |
|  | **Females** | **Males** |
|  | **Mixed effects model**  **(Depression N observations/Total N observations)** | **Mixed effects model**  **(Depression N observations/Total N observations)** |
|  | HR  (95% CI) | HR  (95% CI) |
| **Moray House Test** | 0.94  (0.92 to 0.97)  P<0.001  (n=10618/50383) | 0.93  (0.90 to 0.96)  P<0.001  (n=6163/46577) |
| **Position in family** | 1.00  (0.99 to 1.02)  P=0.646  (n=11303/53589) | 1.01  (0.99 to 1.03)  P=0.228  (n=6510/50016) |
| **Size of family** | 1.02  (1.00 to 1.05)  P=0.116  (n=11301/53582) | 1.03  (0.99 to 1.06)  P=0.113  (n=6509/50009) |
| **Carstairs** | 1.02  (1.00 to 1.04)  P=0.030  (n=3859/32898) | 1.00  (0.98 to 1.02)  P=0.779  (n=2306/30265) |
| **Remote location*** | 1.05  (0.90 to 1.22)  P=0.551  (n=3829/32432) | 1.08  (0.92 to 1.27)  P=0.353  (n=2288/29944) |
| **SIMD – Linear** | 1.23  (1.04 to 1.46)  P=0.018  (n=3836/32572) | 0.98  (0.80 to 1.20)  P=0.839  (n=2291/30077) |
| CI: confidence interval; HR: hazards ratio; SIMD: Scottish Index of Multiple Deprivation | | |

| **Supplementary Table S7:** Risk factors associated with depression after unadjusted and adjusted analyses. Outcome is depression identified in prescribed drugs records only (not hospital admissions data) | | | | |
| --- | --- | --- | --- | --- |
|  | **Unadjusted risk factors** | **Model 1:**  **Adjusted for childhood**  **risk factors** | **Model 2:**  **Adjusted for adulthood**  **risk factors** | **Model 3:**  **Adjusted for all**  **risk factors** |
|  | **Mixed effects model**  **(Depression N observations/Total N observations)** | **Mixed effects model** | **Mixed effects**  **model** | **Mixed effects model** |
| **Depression N/Total N (%)** | N/A | 12214/96102  (13%) | 1225/57341  (2%) | 1219/56771  (2%) |
| **Male** | 0.72  (0.70 to 0.75)  P<0.001  (n=13182/104762) | 0.72  (0.70 to 0.72)  P<0.001 | 1.05  (0.94 to 1.18)  P=0.410 | 1.04  (0.93 to 1.07)  P=0.527 |
| **Moray House Test** | 1.01  (1.00 to 1.03)  P=0.145  (n=12306/96960) | 1.00  (0.98 to 1.02)  P=0.100 | 1.01  (0.95 to 1.07)  P=0.760 | 1.01  (0.95 to 1.07)  P=0.844 |
| **Position in family** | 0.99  (0.98 to 1.00)  P=0.171  (n=13058/103605) | N/A | N/A | N/A |
| **Size of family** | 0.99  (0.97 to 1.01)  P=0.326  (n=13055/103591) | 0.99  (0.97 to 1.01)  P=0.268 | N/A | 0.99  (0.94 to 1.05)  P=0.813 |
| **Carstairs** | 0.97  (0.95 to 0.99)  P<0.001  (n=1345/63163) | N/A | 0.97  (0.95 to 0.99)  P=0.001 | 0.97  (0.95 to 0.99)  P=0.002 |
| **Remote location*** | 1.16  (1.00 to 1.35)  P=0.046  (n=1326/62376) | N/A | 1.12  (0.96 to 1.31)  P=0.151 | 1.13  (0.96 to 1.32)  P=0.140 |
| **SIMD – Linear** | 1.12  (0.99 to 1.28)  P=0.077  (n=1332/62649) | N/A | N/A | N/A |
| CI: confidence interval; HR: hazards ratio; SIMD: Scottish Index of Multiple Deprivation  *Accessible Rural Areas, Remote Rural Areas, Very Remote Rural Areas | | | | |

| **Supplementary Table S8:** Risk factors associated with depression after unadjusted and adjusted analyses. Outcome is depression identified in hospital admissions data only (not prescribed drugs data) | | | | |
| --- | --- | --- | --- | --- |
|  | **Unadjusted risk factors** | **Model 1:**  **Adjusted for childhood**  **risk factors** | **Model 2:**  **Adjusted for adulthood**  **risk factors** | **Model 3:**  **Adjusted for all**  **risk factors** |
|  | **Mixed effects model**  **(Depression N observations/Total N observations)** | **Mixed effects model** | **Mixed effects**  **model** | **Mixed effects model** |
| **Depression N/Total N (%)** | N/A | 4381/96102  (4.6%) | 4412/57341  (7.7%) | 4318/56771  (7.6%) |
| **Male** | 0.78  (0.71 to 0.86)  P<0.001  (n=4861/104762) | 0.78  (0.71 to 0.86)  P<0.001 | 0.79  (0.72 to 0.88)  P<0.001 | 0.79  (0.71 to 0.88)  P<0.001 |
| **Moray House Test** | 0.86  (0.82 to 0.90)  P<0.001  (n=4475/96960) | 0.86  (0.81 to 0.90)  P<0.001 | 0.95  (0.90 to 1.00)  P=0.031 | 0.94  (0.89 to 0.99)  P=0.026 |
| **Position in family** | 1.03  (1.00 to 1.05)  P=0.045  (n=4755/103605) | N/A | N/A | N/A |
| **Size of family** | 1.08  (1.03 to 1.13)  P=0.002  (n=4755/103591) | 1.03  (0.98 to 1.08)  P=0.265 | N/A | 1.00  (0.95 to 1.05)  P=0.910 |
| **Carstairs** | 1.02  (1.01 to 1.04)  P=0.003  (n=4820/63163) | N/A | 1.02  (1.00 to 1.04)  P=0.022 | 1.02  (1.00 to 1.04)  P=0.031 |
| **Remote location*** | 1.04  (0.92 to 1.18)  P=0.535  (n=4791/62376) | N/A | 1.08  (0.94 to 1.23)  P=0.272 | 1.08  (0.95 to 1.23)  P=0.259 |
| **SIMD – Linear** | 1.12  (0.99 to 1.28)  P=0.077  (n=4795/62649) | N/A | N/A | N/A |
| CI: confidence interval; HR: hazards ratio; SIMD: Scottish Index of Multiple Deprivation  *Accessible Rural Areas, Remote Rural Areas, Very Remote Rural Areas | | | | |

**Supplementary Figure S9:** Survival plot showing probability of survival (without depression) in those who had a childhood cognitive test score in the top 2% of the study sample, compared to those who did not score in the top 2% of the study sample.


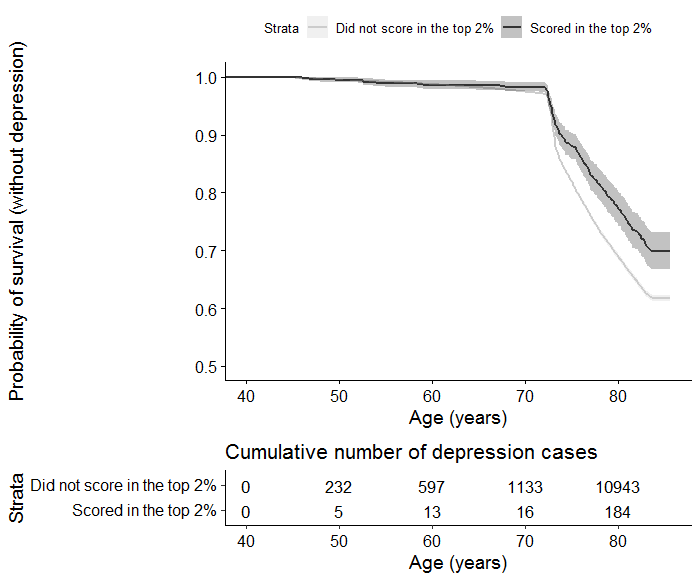


Childhood intelligence test scores were age adjusted, IQ-scaled and standardised.
